# Dynamic changes in ventricular depolarization during exercise in patients with Brugada syndrome

**DOI:** 10.1371/journal.pone.0229078

**Published:** 2020-03-03

**Authors:** Daniel Romero, Nathalie Behar, Bertrand Petit, Vincent Probst, Frederic Sacher, Philippe Mabo, Alfredo I. Hernández

**Affiliations:** 1 Univ Rennes, CHU Rennes, Inserm, LTSI - UMR 1099, Rennes, France; 2 Service Cardiologie, GH Sud. Saint Pierre La Réunion, Saint-Pierre, France; 3 Service Cardiologie, CHU Nantes, Nantes, France; 4 Service Cardiologie, CHU Bordeaux, Bordeaux, France; University of Minnesota, UNITED STATES

## Abstract

Brugada syndrome (BS) is a genetic pathological condition associated with a high risk for sudden cardiac death (SCD). Ventricular depolarization disorders have been suggested as a potential electrophysiological mechanism associated with high SCD risk on patients with BS. This paper aims to characterize the dynamic changes of ventricular depolarization observed during physical exercise in symptomatic and asymptomatic BS patients. To this end, cardiac ventricular depolarization features were automatically extracted from 12-lead ECG recordings acquired during standardized exercise stress test in 110 BS patients, of whom 25 were symptomatic. Conventional parameters were evaluated, including QRS duration, R and S wave amplitudes ($A_{R}$, $A_{S}$), as well as QRS morphological features, such as up-stroke and down-stroke slopes of the R and S waves ($U_{R}$, $D_{R}$ and $U_{S}$). The effects of physical exercise and recovery on the dynamics of these markers were assessed in both BS populations. Features showing significantly different dynamics between the studied groups were used alone and in combination with the clinical characteristics of the patients in a logistic regression analysis. Results show larger changes in the second half of the QRS complex through $A_{S}$ and $U_{S}$ measured in the right precordial leads for asymptomatic patients, especially during recovery, when the vagal tone is more pronounced. Multivariate analysis involving both types of features resulted in a reduced model of three relevant features ($\Delta A_{S}$ in lead V2, *Sex* and heart rate recovery, *HRR*), which achieved a suitable discrimination performance between groups; sensitivity = 80% and specificity = 75% (AUC = 83%). However, after controlling the model for possible confounding factors, only one feature ($\Delta A_{S}$) remained meaningful. This adjusted model significantly improved the overall discrimination performance by up to: sensitivity = 84% and specificity = 100% (AUC = 94%). The study highlights the importance of physical exercise test to unmask differentiated behaviors between symptomatic and asymptomatic BS patients through depolarization dynamic analysis. This analysis together with the obtained model may help to identify asymptomatic patients at low or high risk of future cardiac events, but it should be confirmed by further prospective studies.

## Introduction

The Brugada syndrome (BS) is a genetic pathology associated with a high risk for sudden cardiac death (SCD) in patients with apparent structurally normal heart [1, 2]. Brugada syndrome is diagnosed when ST-segment elevation ≥2 mm (type 1 morphology) is present in ≥1 lead in the right precordial leads V1, V2, positioned in the 2*^nd^*, 3*^rd^*, or 4*^th^*, intercostal space, occurring either spontaneously or after provocative drug test with intravenous administration of Na^+^ channel blockers [1, 2]. According to the most recent international guidelines [1], the implantation of a cardioverter-defibrillator (ICD) is recommended (class I) in patients with a diagnosis of BS who are survivors of an aborted cardiac arrest and/or have documented spontaneous sustained VT and should be considered (class IIa) in patients with a spontaneous diagnostic type I ECG pattern and history of syncope. However, the decision of implanting an ICD is more complex on asymptomatic patients, representing around 60% of the patients diagnosed with BS, since they have a much lower risk of arrhythmic events, recently estimated at less than 1% on the FINGER study [3]. Moreover, during a long-term follow-up, around 30% of implanted asymptomatic patients have suffered their first appropriate ICD shock in 10 or more years after the implantation time [4]. Likewise, the incidence of inappropriate ICD-shocks and other device-related complications increases with longer follow-up duration, mostly affecting young and active people with a live expectancy ≥ 30 years. Thus, one of the main challenges today is to better identify which of the asymptomatic patients might benefit from an ICD implantation, using an appropriate risk stratification tool.

A number of depolarization disorders have been observed on BS patients, including a high prevalence of QRS late potentials (LP), fragmented QRS complexes [5], prolongation of PR/QRS duration [6], wider S waves in inferolateral leads and rightward deviation of the axis in the terminal quarter of the QRS loop [7]. Moreover, the autonomic nervous system (ANS) function has been reported to have a significant role in unmasking differences in this pathology, particularly during exercise testing. Amin et al. [8] reported that exercise resulted in an increase of J-point amplitude in both BS and control groups. Makimoto et al. [9] evaluated the relationship between the post stress parasympathetic activation and the ST-segment change. It has also been reported that parasympathetic reactivation during early recovery, assessed through the heart rate recovery (HRR) index, tends to be higher only in BS patients with prior ventricular fibrillation (VF) episodes [8].

Recent works from our group have shown that symptomatic BS patients exhibit greater fluctuations in sinus node response to ANS in 24-h Holter recordings [10] and an increased parasympathetic modulation during incremental exercise and early recovery [11]. However, these results were mainly focused on the analysis of ANS response according to patients’ symptomatology. The inherent multifactorial nature of BS requires to consider other major players involved in the pathogenesis of the disease beyond the ANS. For instance, multivariate methods that combine electrophysiological markers and traditional risk factors have recently demonstrated their usefulness for assessing the VF risk in BS patients [12]. Moreover, the importance of multivariate approaches has been valued in [13], where the authors refer to major studies conducted in this context and suggest them as the future in BS. Therefore, the assessment of non-invasive risk markers using electrophysiological parameters in conjunction with autonomic-related markers during controlled autonomic maneuvers, appears to be of particular importance.

In this study, we propose a complementary analysis with respect to our previous works, in which advanced signal-processing methods are applied to extract quantitative markers of ventricular depolarization during exercise stress tests in patients with BS. The dynamic changes of these electrophysiological markers, which are associated with ventricular electrical conduction velocity, were then analyzed to assess differences between symptomatic and asymptomatic BS patients. Clinical characteristics of the population as well as the HRR index were finally integrated into a combined multivariate model to improve discriminative performance among these two groups of patients.

## Materials and methods

### Population

110 consecutive patients (85 males) diagnosed with BS were enrolled in a prospective, multicentric study, led by the Cardiology department of the Rennes University Hospital (CHU de Rennes) between 2008 and 2013, in France. It was conducted in accordance with applicable good clinical practice requirements in Europe, French law, ICH E6 recommendations, and the ethical principles of the Helsinki Declaration (1996 and 2000). Participants were enrolled in 6 French hospitals located in Rennes, Saint Pierre de la Réunion, Nantes, Bordeaux, Brest and La Rochelle. The study protocol was approved by the respective local ethics committees: Comité d’Éthique du CHU de Rennes (ID RCB 2007-A00887-46), Comité d’Éthique du CHU Saint-Pierre, Comité d’Éthique du CHU de Nantes, Comité d’Éthique du CHU de Bordeaux, Comité d’Éthique du CHU de Brest and Comité d’Éthique du Centre Hospitalier de La Rochelle. All patients provided their written informed consent before participation.

Patients mean age was 44.6±13.7 years. BS was diagnosed when a coved ST-segment elevation (≥0.2 mV) was spontaneously observed in ≥1 right precordial leads located in the 2*^nd^*, 3*^rd^* or 4*^th^* intercostal space, or induced during a sodium channel-blocking administration, according to the current guidelines [1, 2]. Twenty-five patients experienced syncope or aborted SCD related to VF, near syncope and palpitations. These patients were classified as symptomatic. The remaining 85 patients were thus classed as asymptomatic. Structural heart disease was excluded based on physical examination, patient’s history, resting and exercise ECG. Likewise, no significant left ventricular hypertrophy was observed on any patient during echocardiographic screening. ICDs had been implanted in 44 (38%) patients including the whole symptomatic group, and those asymptomatic patients who had a positive EPS (Electrophysiological Study) test for arrhythmia inducibility. From the total group, only 79 patients underwent a genetic testing, of whom 29 (7 symptomatic) were positive for the SCN5A mutation. The other patients were not genetically screened because no mutations were identified in their families. In this study, only SCN5A mutations were screened, according to the active guidelines and practice at the time of the protocol definition, in 2008.

Stress Test Protocol: A standard 12-lead ECG acquired at a sampling rate of 1000 Hz was recorded for each patient using a standardized exercise stress test performed on a cycle ergometer (Ergoline 900 Egamed, Piestany, Slovakia), defined by the following protocol:

- Exercise period (EX): Comprised of an initial workload of 2 minutes pedaling at 50 W (30 W for women) and followed by successive increments of 30 W (20 W for women) every 2 minutes, until the patient reached at least 80% of the theoretical maximum HR (*HR*_*max*_ = 220–age).
- Recovery period (RE): It included two successive three-minute periods, one of active recovery with the patient pedaling at a workload of 50 W, followed by a passive recovery at rest.

### Data preprocessing

All ECG signals were preprocessed before the automatic extraction of the analyzed indices. This step included automatic QRS complex detection [14] and subsequent visual inspection to avoid inclusion of abnormal beats, baseline drift attenuation via cubic spline interpolation, 4-th order bidirectional Butterworth low pass filtering at 45 Hz to remove high frequency (e.g., muscular noise) noise and, wave boundaries delineation using an evolutionary optimization approach [14].

### Cardiac depolarization features extraction

#### Conventional depolarization markers

Conventional ECG markers associated with ventricular depolarization were assessed from the ECG signals: the amplitude of the R and S waves ($A_{R}$, $A_{S}$) and QRS duration (*QRSd*). *QRSd* was calculated by using a multilead-based approach that determines the earliest QRS onset, and latest QRS end among the available ECG leads [15]. The above approach was applied to all precordial leads, V1-V6, while both the $A_{R}$ and $A_{S}$ markers were extracted only from leads V1-V3.

#### Novel depolarization markers

**Fig 1:**
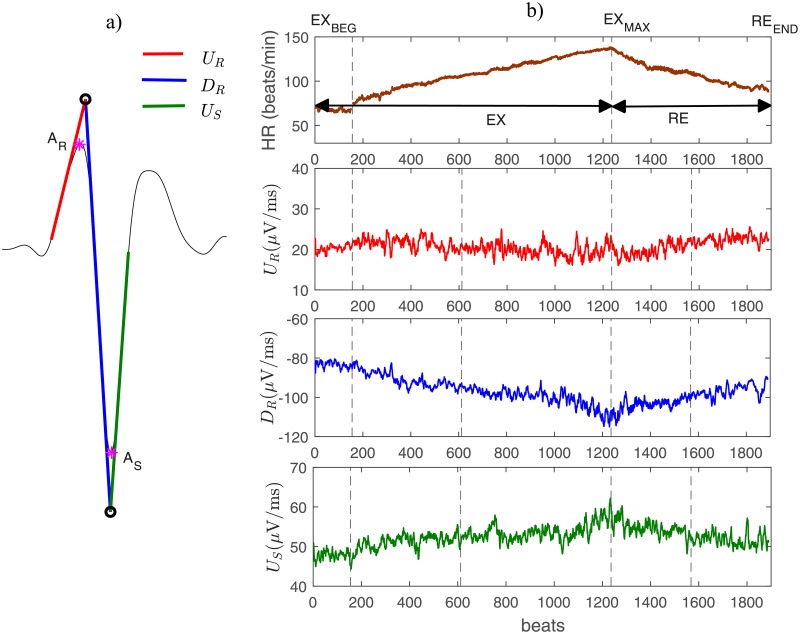
A) Example of a QRS complex and the three QRS slopes analyzed in this study (red, blue and green lines). B) Temporal evolution of heart rate (upper panel) and the main extracted ECG markers (panels 2 to 4) evaluated in a representative BS patient during the whole exercise test. The major periods of the exercise test are marked in the upper panel of Fig 1B: exercise period (EX); recovery period (RE). Panel 1 also shows the phases at which the mean values were determined: the beginning of the exercise (EX_beg_); the time of maximum effort (EX_max_); the end of the entire recovery period (RE_end_).

In addition to the above-mentioned conventional markers, three morphological markers were also extracted from ECG signals (see Fig 1a): the upstroke (red line) and downstroke (blue line) slopes of the R wave (noted $U_{R}$, and $D_{R}$, respectively) and the terminal upstroke slope (green line) of the S wave ($U_{S}$). These markers are the result of fitting a straight line over the ECG signal in specific segments within the QRS complex as described in [16]. Fig 1B shows the evolution of heart rate and three of the proposed depolarization markers, throughout the whole exercise stress test, for a representative patient.

### Depolarization dynamics analysis

**Fig 2:**
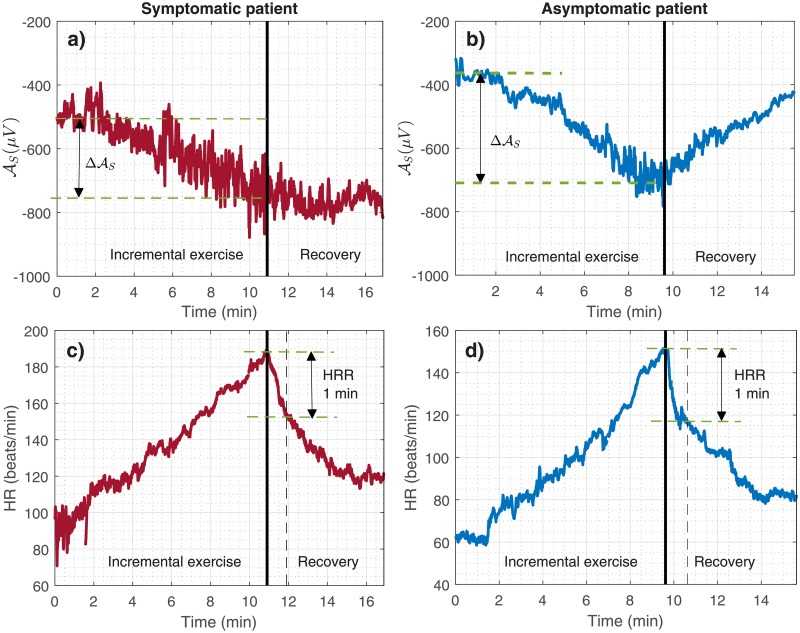
a) and c) Temporal evolution of the S-wave amplitude ($A_{S}$) and heart rate (HR) during the exercise test for a male symptomatic patient; b) and d) for a male asymptomatic patient. The heart rate recovery (HRR) estimate and the change of $A_{S}$ during exercise ($\Delta A_{S}{}_{\text{EX}}$) are also shown. Thick vertical lines in black indicate the time of maximum effort (EX_max_).

In order to analyze the dynamics of cardiac depolarization during effort and recovery, mean values of the depolarization markers were first obtained at different phases of the exercise test. These phases were defined as follows (see Fig 1b): 1) the beginning of the exercise test or baseline exercise phase (EX_beg_); 2) the time of maximum effort (EX_max_); 3) and the end of the complete recovery period, including both the active and passive recovery periods (RE_end_). The mean values were calculated in 15-s windows duration for each phase. Throughout the manuscript, these values will be referred as $\bar{Y}$, where $Y\in \{QRS_{d},A_{R},A_{S},U_{R},D_{R},U_{S}\}$. Dynamic changes, expressed as ΔY, were subsequently assessed as the difference observed during a transition period defined by two specific phases from those previously described. In general, these changes can be expressed as: $\Delta Y_{i,j}=Y_{i}-Y_{j}$, in which *i* and *j* represent the involved phases. Two transition periods were thus analyzed: i) between the EX_beg_ and EX_max_ phases, indicating the change occurred during the effort (denoted $\Delta Y_{\text{EX}}$) and, ii) between the EX_max_ and RE_end_ phases (denoted $\Delta Y_{\text{RE}}$), indicating the change occurred during the whole recovery period. Fig 2 shows the evolution of $A_{S}$ and its associated $\Delta A_{S}{}_{\text{EX}}$, for one representative male patient of the symptomatic (Fig 2a) and asymptomatic (Fig 2b) groups.

### Heart rate recovery

The heart rate recovery index (*HRR*), an autonomic-related measure resulting from a combination of the vagal activation and sympathetic withdrawal during the recovery period after effort, was also assessed in this study [17]. *HRR* is defined as the heart rate decay occurred during the first minute of recovery evaluated immediately after maximal effort, as illustrated in Fig 2c and 2d for two representative patients.

### Statistical analysis

Results were expressed as mean ± SD. Statistical analysis was carried out using the Wilcoxon rank-sum non-parametric test when comparing the inter-group differences. To compare categorical variables, the Pearson’s chi-square test ($X^{2}$ test) was used. In all the analyses the level of significance was set to 0.05.

The depolarization markers showing statistically significant differences between groups during the univariate analysis were used as input to a multivariate logistic regression analysis (MLRA). Two MLRA models were created: a first model (**Model**_**1**_) integrating only selected ventricular depolarization markers and a second model (**Model**_**2**_) integrating ventricular depolarization markers and clinical features.

The LASSO L1-regularization technique was applied in both cases to find the most predictive features, while protecting against overfitting [18]. This method makes possible to obtain sparse models and thereby to better interpret the final outcomes. By varying the regularization strength λ, different subsets of predictive variables can be selected. The larger the λ, the smaller the number of selected variables. However, there is an optimal λ (the best predictive model) which is found with the smallest mean cross-validated error during the learning process. Nevertheless, any other model whose error is within one standard error (SE) around the best model would also be a suitable choice. Therefore, a sparse solution (**Model**_**1**_ and **Model**_**2**_) meeting the above criterion was selected by splitting the entire population in 10 folds, and performing the cross-validation process that generates 10 testing errors, obtained from different, equally split population subsets not seen during learning.

The features selected according to the optimal λ were then used to obtain the final model applied to the whole dataset. In this last step, since standard statistical inference cannot be applied to LASSO coefficients, logistic regression was conducted without regularization, allowing for the detection of the most significant characteristics and further reducing the initial subset of predictive characteristics, yielding to even more reduced models denoted as **Model**_**XR**_, with **X** = {1, 2}. Moreover, potential confounding factors such as SNC5A mutation, ICD data and BMI values, were used for correcting the models, which were defined as ${\text{Model}}_{\text{XR}}^{\text{factors}}$. Finally, receiver operating characteristics (ROC) curves were generated for all models to assess sensitivity, specificity and the area under the ROC curve (AUC).

## Results

### Population characteristics

Table 1 summarizes the main clinical characteristics of the BS population investigated in this study. It also includes the inter-group comparison, showing no significant differences among them in all features.

**Table 1:** Baseline clinical characteristics of the Brugada syndrome population at the time of diagnosis.

Clinical Characteristic	Total	Symptomatic	Asymptomatic	p-value
*Sex* males	82 (74%)	21 (84%)	61 (72%)	0.22
*Age* (years)	44.6±13.7	46.8±15.5	44.0±13.1	0.63
Symptoms	25 (22%)			
Cardiac arrest		11 (44%)		
Syncope		14 (56%)		
Spontaneous Type-1 *ECG*	34 (30%)	7 (28%)	27 (32%)	0.72
ICD implanted	44 (38%)	25 (100%)	19 (22%)	< 0.01
SNC5A mutation (79 pats.)				
Positive	29 (26%)	7 (28%)	22 (26%)	
Negative	50 (44%)	13 (52%)	37 (44%)	0.85
Non-tested	31 (30%)	5 (20%)	26 (30%)	
Maximum HR, *HR*_*max*_ (beats/min)	158.8±18.6	151.7±17.5	160.9±18.5	0.07
Maximum workload (Watts)	170.4±57.6	171.2±57.8	170.1±57.9	0.66
*HRR* (beats/min)	20.9±8.7	18.0±8.8	21.7±8.6	0.25
*BMI* (kg)	71.4±14.7	75.4±12.5	70.2±15.2	0.07

### Univariate analysis

**Fig 3:**
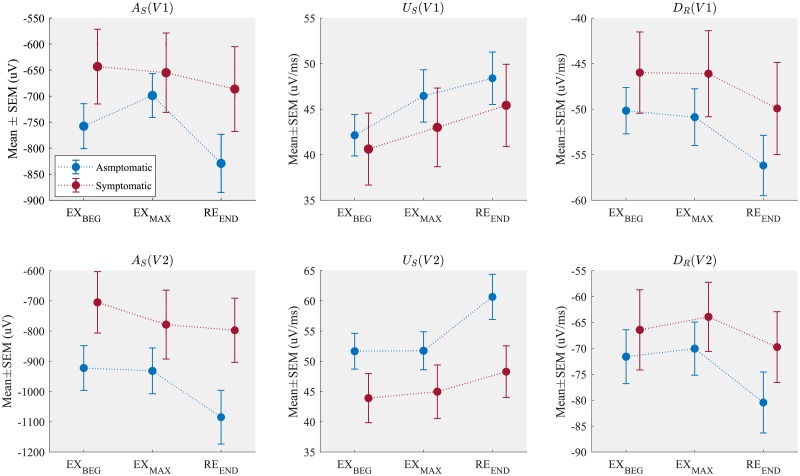
Mean ± SEM of the amplitude of the S wave ($A_{S}$), the upstroke slope of the S wave ($U_{S}$) and the downstroke slope of the R wave ($D_{R}$), measured in different phases of the exercise test in leads V1 and V2.

Fig 3 shows the mean values of $A_{S}$, $U_{S}$ and $D_{R}$ computed at different stages of the exercise test for each patient group. Asymptomatic patients presented a larger S wave deflection (lower S wave amplitude values) as well as steeper upstroke and downstroke QRS slopes, when compared to the symptomatic group in all phases, even when it was measured at baseline (EX_beg_). Moreover, the asymptomatic group showed significant variations from the maximal effort to the end of recovery, while little or negligible changes were observed during incremental exercise, particularly in lead V2. A complete table of the observed values of each marker is presented in the S1 Table.

Dynamic changes quantified between the exercise and the recovery periods for all markers are presented in Table 2. Concerning the comparison between symptomatic and asymptomatic patients during incremental exercise, only $A_{S}$ as observed in lead V1 showed significant differences, being the dynamics of this marker higher for asymptomatic patients. Regarding the comparison between symptomatic and asymptomatic patients during recovery, changes in $A_{S}$, $U_{S}$ and $D_{R}$ were significantly different between both populations for leads V1-V3. More precisely, the asymptomatic group presented greater absolute values (greater dynamics) as compared to symptomatic patients. The remaining markers did not show statistically significant changes among groups, despite of the fact that their values were slightly higher in asymptomatic patients.

**Table 2:** Dynamic changes of depolarization markers between the exercise and recovery periods for symptomatic and asymptomatic patients. Results are expressed in mean±SD. Statistically significant *p*-values were highlighted as follows: when comparing both groups during exercise, *(*p* < 0.05); when comparing both groups during recovery, † (*p* < 0.05), ‡(*p* < 0.01),§ (*p* < 0.005).

ECG index	Symptomatic	Asymptomatic	Symptomatic	Asymptomatic
	Exercise	Exercise	Recovery	Recovery
$\Delta A_{R}(\mu \text{V})$				
V1	30±52	83±357	-10±66	-63±354
V2	-57±142	-57±132	29±94	47±113
V3	-27±78	-104±198	34±136	92±174
$\Delta A_{S}(\mu \text{V})$				
V1	-12±120	59±258*	-31±158	-130±295^§^
V2	-73±172	-9±179	-19±206	-153±227^‡^
V3	-259±196	-239±229	50±202	9±227
$\Delta U_{S}(\mu \text{V}/\text{ms})$				
V1	2±9	4±14	2±9	2±13
V2	1±9	0±8	3±10	9±11^§^
V3	8±9	7±9	-1±8	3±10^†^
$\Delta D_{R}(\mu \text{V}/\text{ms})$				
V1	0±6	-1±15	-4±10	-5±13
V2	3±15	2±12	-6±13	-10±14
V3	-16±16	-10±16	3±19	-3±18^†^
$\Delta U_{R}(\mu \text{V}/\text{ms})$				
V1	3±5	6±23	-2±5	-4±21
V2	-1±7	0±8	0±6	1±6
V3	5±7	2±10	-2±8	0±10
Δ*QRSd* (ms)	2±17	6±21	0±15	-6±19

### Multivariate analysis using clinical and depolarization features

By using the most relevant variables reported in Table 2, **Model**_**1**_ was obtained with the predictive parameters subset selected with the LASSO approach. Table 3 summarizes the main properties of this sparse model which is based solely on depolarization dynamic features. According to the results, only four features were retained in this model, of whom two were significant. The negative sign of the coefficients and the odds ratios (OR) values suggest that the significant parameters act as protective factors rather than symptom-based risk factors (OR<1).

**Table 3:** Model_1_: Logistic regression model using depolarization features selected by Lasso *L1*-regularization.

Model	Features	Coefficients	OR (95% CI)	p-value
**Model**_**1**_: Depolarization features	Constant	4.76	118 (1.77–7951)	0.026*
	$\Delta U_{S(RE)}\;(\text{V}2)$	-1.73	0.18 (0.01–3.37)	0.249
	$\Delta A_{S(EX)}\;(\text{V}2)$	-3.42	0.03 (0.00–0.98)	0.048*
	$\Delta A_{S(EX)}\;(\text{V}3)$	-4.62	0.01 (0.00–0.78)	0.038*
	$\Delta U_{S(EX)}\;(\text{V}3)$	-3.08	0.05 (0.00–1.92)	0.106

To assess the added value of depolarization dynamic features, **Model**_**2**_ was obtained following the same strategy as for **Model**_**1**_. In this case, the clinical characteristics reported in Table 1 were added as input variables in the LASSO-based selection step, excluding genetic screening and ICD data. Table 4 presents the summary of this second model after it was trained with the entire population.

**Table 4:** Model_2_: Logistic regression model using clinical and depolarization features selected by Lasso.

Model	Features	Coefficients	OR (95% CI)	*p*-value
**Model**_**2**_: Depolarization features + Clinical features	*Constant*	4.76	371 (3.97–34739)	0.010*
	*Sex*	1.73	5.67 (1.31–24.5)	0.020*
	Type-1 *ECG*	-0.39	0.67 (0.20–2.22)	0.514
	$\Delta U_{S(RE)}\;(\text{V}2)$	-1.91	0.15 (0.01–3.39)	0.232
	$\Delta A_{S(EX)}\;(\text{V}2)$	-4.83	0.01 (0.00–0.39)	0.015*
	$\Delta A_{S(EX)}\;(\text{V}3)$	-1.94	0.14 (0.01–3.68)	0.241
	$HR_{\text{MAX}}$	-3.32	0.04 (0.00–1.09)	0.056
	*HRR*	-2.89	0.06 (0.00–0.93)	0.044*
	*Age*	-1.99	0.14 (0.01–3.34)	0.222

*p<0.05

**Fig 4:**
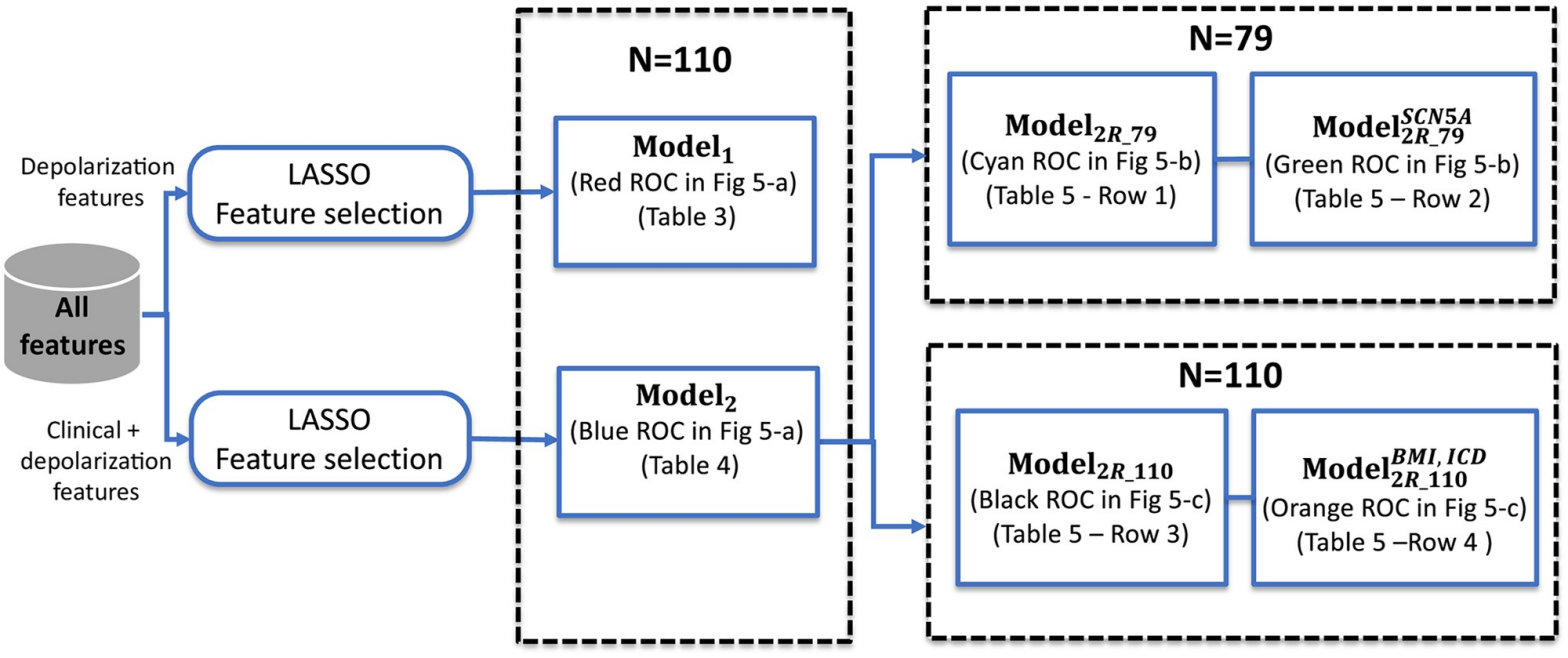
Flowchart diagram of the processing pipeline used to obtain the different models investigated in the study.

As it can be seen from Table 4, eight features, including three that were included in **Model**_**1**_, were retained for this combined model. The most relevant features were $\Delta A_{S(EX)}$ in lead V2, *Sex* and *HRR*, as reflected by their *p*-values. Concerning $\Delta A_{S(EX)}$ and *HRR*, negative coefficients indicate that for each one-unit increase in these features, the risk of being symptomatic decrease by a 0.01 and a 0.06-fold, respectively. This means that one-unit increase in $\Delta A_{S(EX)}$ and *HRR*, increases the odds of not being symptomatic over 100-fold and 16.7-fold, respectively (1/0.01 = 100 and 1/0.06 = 16.7). Finally, concerning the *Sex* variable, the OR = 5.67 means that men have 5.67-fold higher risk of being symptomatic patients than women. By using only these three significant features, further reduced models were considered bellow, following the flowchart presented in Fig 4.

Because the genetic screening was not performed in all patients, the multivariate analysis was separately applied to the subgroup of patients for which SNC5A mutation information was available (*N* = 79). Only significant parameters in Table 4 were included in the model, denoted as **Model**_**2R_79**_. For this smaller subgroup, all markers remained significant, with *Sex* having an OR of 13.33 (*p* = 0.036), while for *HRR* it was <0.01 (*p* = 0.003) and 0.01 (*p* = 0.010) for $\Delta A_{S(EX)}$. Indeed, *p*-values associated with *HRR* and $\Delta A_{S(EX)}$ were smaller than those of Table 4. Afterwards, the model was adjusted for the mutation data as a potential confounding factor, and results were not significantly affected as it is shown in Table 5 (see ${\text{Model}}_{2\text{R}\_79}^{\text{SCN}5\text{A}}$), except for *Sex*, whose OR increased from 13.3 to 20.6 (>50%). This suggests that association between male gender and symptoms is enhanced by the presence of the SCN5A mutation.

**Table 5:** Reduced models using only significant features from Table 4, before and after adjusting by confounding factors. Only patients that underwent genetic screening were included (*N* = 79) in the first two models. The last two models included the whole population study (*N* = 110).

Model	Features	Coefficients	OR (95% CI)	*p*-value
**Model**_**2R_79**_: Significant features	*Constant*	1.93	0.69 (0.03–16.52) ×10^1^	0.234
	*Sex*	2.59	1.33 (0.12–14.86) ×10^1^	0.036*
	*HRR*	−4.40	0.01 (0.00–0.14)	0.010*
	$\Delta A_{S(EX)}\;(\text{V}2)$	−5.67	0.34 (0.00–14.03) ×10^−2^	0.003*
${\text{Model}}_{2R\_79}^{\text{SCN}5A}$: Significant features + mutation data	*Constant*	1.43	0.42 (0.02–10.83) ×10^1^	0.389
	*Sex*	3.02	2.06 (0.15–27.39) ×10^1^	0.020*
	*HRR*	−4.48	0.01 (0.00–0.31)	0.008*
	$\Delta A_{S\left(EX\right)}\;(\text{V}2)$	−5.97	0.25 (0.00–11.35) ×10^−2^	0.002*
	SCN5A^(+)^	0.86	2.37 (0.59–9.49)	0.222
**Model**_**2R_110**_: Significant features	*Constant*	1.67	5.29 (0.66–42.49)	0.117
	*Sex*	1.49	4.45 (1.13–17.52)	0.033*
	*HRR*	−2.99	0.05 (0.00–0.57)	0.016*
	$\Delta A_{S(EX)}\;(\text{V}2)$	−5.10	0.01 (0.00–0.16)	0.002*
${\text{Model}}_{2R\_110}^{\text{BMI},\text{ICD}}$: Significant features + BMI and ICD data	*Constant*	−100.61	2.03×10^44^ (0.00–1×10^99^))	0.999
	*Sex*	−0.14	0.87 (0.11–6.66)	0.893
	*HRR*	−0.55	0.58 (0.02–20.88)	0.763
	$\Delta A_{S\left(EX\right)}\;(\text{V}2)$	−6.22	0.19 (0.00–30.68) ×10^−2^	0.016*
	*BMI*	2.77	16.05 (0.14–1870.50)	0.253
	*ICD*	102.99	5.33×10^44^ (0.00–1 ×10^99^)	0.999

*p<0.05

Since mutation data had no significant impact on two features of the reduced model, and the association of *Sex* with symptoms was strengthened rather than penalized, other possible confounding factors such as *BMI* and ICD data were investigated to adjust the same model, but now using the whole initial population (*N* = 110), including thus patients for which no genetic screening was performed. Unlike the unadjusted model, **Model**_**2R_110**_, only $\Delta A_{S(EX)}$ remained significant among the three involved features (see ${\text{Model}}_{2R\_110}^{\text{BMI},\text{ICD}}$ in Table 5). For instance, $\Delta A_{S(EX)}$ had an OR of 0.006 (*p* = 0.002) and its protective role was reinforced for the adjusted model (OR = 0.002, *p* = 0.016). Conversely, *BMI* and ICD data confound the role of *Sex* (OR = 4.45, *p* = 0.033) as a potential risk factor as well as the protective role of *HRR* (OR = 0.05, *p* = 0.016) in terms of symptoms association, which were no longer meaningful after adjustment. Statistics about these two models are summarized in Table 5.

**Fig 5:**
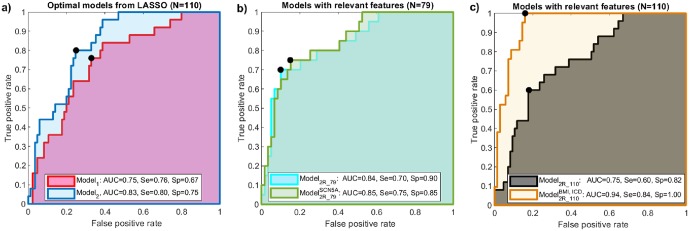
a) Receiver operating characteristic (ROC) curves obtained for the two discriminative models summarized in Table 3 (**Model**_**1**_) and Table 4 (**Model**_**2**_), (b) for the reduced models adjusted (${\text{Model}}_{2R\_79}^{\text{SCN}5A}$) and non adjusted (**Model**_**2R_79**_) by mutation data, applied on the screened 79 patients, and c) for the reduced model using significant features from Table 3, adjusted (${\text{Model}}_{2R\_110}^{\text{BMI},\text{ICD}}$) and non adjusted (**Model**_**2R_110**_) by BMI and ICD data. Solid circles in black represent the optimal operating points determining the sensitivity and specificity values from each ROC. AUC: area under the ROC curve (coloured areas); Se: sensitivity; Sp: specificity.

Fig 5a shows the comparison between the two optimal models (obtained from LASSO) in terms of discrimination performance. ROC curves and their associated AUC values are displayed. Sensitivity and specificity were determined from the points on the ROC closest to the upper-left corner. Results show that the model combining clinical and depolarization features (**Model**_**2**_—Table 4) provided slightly better results than the model based solely on depolarization parameters (**Model**_**1**_—Table 3). Note that **Model**_**1**_ may still be suitable for discriminating between BS patient groups, using half the number of parameters with respect to **Model**_**2**_. Performance metrics remained similar when non significant features in Table 4 were removed from **Model**_**2**_, in order to create the even more reduced model (**Model**_**2R_110**_). However, this reduced model with only 3 features, tested on the population subset that underwent genetic screening (*N* = 79), presented similar overall performances before (**Model**_**2R_79**_) and after (${\text{Model}}_{2R\_79}^{\text{SCN}5A}$) adjusting for the presence/absence of the mutation, as shown in Fig 5b. Finally, when **Model**_**2R_110**_ was adjusted by BMI and ICD data (${\text{Model}}_{2R\_110}^{\text{BMI},\text{ICD}}$), these performance metrics were significantly improved (see Fig 5c), with *AUC* = 94%, *Sp* = 84% and *Se* = 100%).

## Discussion

Stratifying the level of risk in asymptomatic Brugada patients is still a major clinical challenge. Better discriminative markers are needed to improve prognosis and to optimize the therapy for a given patient. Apart from classical high-risk markers reported in previous BS-related studies, such as spontaneous type-I ECG pattern and VF history, the analysis of ventricular depolarization in this population may provide an interesting source of information, especially if combined with ANS-related parameters. In fact, it has been previously shown that the application of multivariate methods that combine clinical and electrophysiological analysis provide more robust estimations for risk stratification in BS [19, 20].

Regarding depolarization analysis, many previous studies have reported noninvasive risk markers for arrhythmic events in patients with BS, related to the QRS complex. For instance, a longer QRS duration in symptomatic patients have been observed in lead V2 [21]. In fact, prolonged QRS duration has been associated with increased risk of cardiac events [21, 22], and values >120 ms were shown to predict ventricular arrhythmia and/or syncope [23]. Likewise, late potentials on signal-averaged ECG seem to be more common in symptomatic patients [24], but with limited prognostic value if considered alone. QRS fragmentation, expressed as multiple spikes within the QRS complex in leads V1-V3, was confirmed in the PRELUDE study as an independent predictor of arrhythmias [25]. The presence of the aVR sign (R-wave amplitude ≥ 0.3 mV or R/q ≥ 0.75 in lead aVR, may reflect more right ventricular conduction delay and consequently increased risk for development of arrhythmic events [26]. Finally, wider and/or large S-wave upstroke (≥ 0.1 mV and ≥ 40 ms, respectively) in lead I, associated with delayed activation in the RVOT, was reported as a powerful predictor of ventricular arrhythmias [27].

In our study, we have investigated the potential usefulness of more robust and refined markers of ventricular depolarization, mainly related to conduction velocity and associated with the R and S waves shape. Unlike some previous studies [21, 28], we did not find any statistical difference in QRS duration between symptomatic and asymptomatic groups. However, significant differences were found for indices $A_{S}$, $D_{R}$ and $U_{S}$ in the right precordial leads V1-V3 and mostly during recovery. These indices presented smaller values in symptomatic patients, and these findings may be associated with those reported in [27] about delayed depolarization in the RVOT. Likewise, results obtained from $A_{S}$, $U_{S}$ (second half of the QRS complex) may be correlated with those obtained from the analysis of late potentials [5]. The role of slow conduction as key indicator of SCD in BS has been supported by the observation of electroanatomic maps of the RV, where patients with recurrent VF episodes showed a prominent delayed depolarization accompanied with low voltage and fractionated electrograms over the anterior epicardial region of the RVOT [6, 29]. Indeed, when radio-frequency ablation of epicardial sites displaying late potentials in the RVOT is applied, the arrhythmic risk and ECG characteristics of BS disappear or are significantly reduced [30]. This fact supports the hypothesis that the elimination of the sites presenting slow conduction may be the basis for the ameliorative effect of ablation therapy. Nevertheless, there is a competing hypothesis regarding the underlying mechanisms of late potentials and fragmented electrograms, based on results from a coronary-perfused canine RV wedge model of BS [31]. In that model, fractionated electrical activity was observed in RV epicardium as a consequence of heterogeneities in the appearance of the second upstroke of the epicardial AP, while discrete high-frequency spikes were a result of concealed phase-2-reentry.

The analysis of the dynamics of ventricular depolarization markers along the main phases of a stress test is another contribution of this work. In general, the major differences found between the two patient groups were associated with significantly larger modifications of depolarization markers through time (depolarization dynamics), observed in asymptomatic patients with respect to the symptomatic group. This large dynamicity in the asymptomatic group was even more pronounced during recovery, especially for markers $A_{S}$ and $U_{S}$, which might be related to the results reported by Makimoto *et al*. [9]. In that study, augmented ST-elevation and peak J-point amplitude during early recovery (1-4 minutes) after exercise cessation, were associated with poor prognosis in patients with syncope alone or asymptomatic subjects. Specifically, changes occurring in the peak J-point amplitude, considered as a depolarization parameter or at least combined parameter of both depolarization and repolarization, can directly affect the S-wave upstroke $U_{S}$ evaluated in this study. Therefore, these findings postulate the exercise testing as a useful tool to unveil distinct electrophysiological responses in BS patients, trough the vagally-mediated accentuation of ECG patterns (i.e., J waves and ST elevation) that may contribute to arrhythmia initiation owing to decreasing *I*_*Ca*_ [32]. The above is supported by the higher incidences of cardiac events at rest or during sleep in BS patients, when the vagal tone is normally increased as it happens during recovery effort.

In addition to the relevant parameters found when exploiting only the ventricular depolarization markers, the clinical characteristics of the population were also included in a multivariate logistic regression analysis. While none of the clinical parameters were significantly different between groups (see Table 1), their combination with depolarization dynamic markers yielded an optimal model of only eight features. This combined model (**Model**_**2**_) was able to discriminate among groups reaching a suitable performance outcome (AUC = 83%, Se = 80%, Se = 75%). Nevertheless, when only depolarization features were used, the performance of the model (**Model**_**1**_) was slightly smaller, despite the fact that only four features were included (AUC = 75%, Se = 76%, Se = 67%). This highlighted the significant contribution of dynamic depolarization properties in terms of discrimination between symptomatic and asymptomatic patients. Further multivariate analyses performed on the smaller subgroup screened for SCN5A mutations, and using only significant features from **Model**_**2**_, showed similar results even after correcting by mutation data. Indeed, this characteristic did not have a strong impact on the overall performance of the model since all the involved features remained significant after adjustment. Only the sex’s association with the presence of symptoms was, in fact, positively impacted (see **Model**_**2R_79**_ and ${\text{Model}}_{2R\_79}^{\text{SCN}5A}$ in Table 5). Lastly, the above reduced model was tested on the whole population excluding mutation data, but considering other possible confounding factors. Interestingly, after correcting for the BMI and ICD data (${\text{Model}}_{2\text{R}\_110}^{\text{BMI},\text{ICD}}$), the model performance increased significantly, reaching metrics of: *AUC* = 94%, *Se* = 84% and *Sp* = 100%. In that model, only one of the three relevant features has remained significant, and it was related to the exercise-related dynamic of $A_{S}$ in lead V2, whose association with the absence of symptoms was strengthened through reinforcement of its protective role. The main outcome of the multivariate analyses was that most significant parameters were, in fact, protective factors rather than risk factors. Therefore, they could serve as potential indicators of low-risk, especially in asymptomatic patients. The only marker that apparently was thought to be a major risk factor was the patient’s sex (see **Model**_**2R_110**_). However, its association with the symptoms was dampened after controlling the model for potential confounding factors.

It is well-known that early recovery of the heart rate, occurring immediately after cessation of exercise, is due to parasympathetic reactivation. Such reactivation is somehow captured by the HRR marker, that turned to be a protective factor in our combined model before adjustment. To some extent, this finding may be linked to the results reported in [11], where a distinct parasympathetic activity was found among BS patients’ groups during incremental exercise and early recovery. Nevertheless, its role as a protective factor was largely affected when controlling for BMI and ICD data.

Finally, as suggested by Postema et al. [6], repolarization abnormalities in Brugada syndrome are mostly induced by depolarization abnormalities. The latter were thought to be a result of the heterogeneity in the action potential duration with a ventricular endo-epi gradient [33]. However, in Meijborg et al. [34], repolarization abnormalities were thought to be related to an increased interventricular and LV-intraventricular dispersion in repolarization time, after dofetilide infusion in an experimental model of dofetilide-induced long QT syndrome type 2 (LQT2). Tokioka et al. [35] reported that combination of both repolarization and depolarization abnormalities enables potential identification of high- and low-risk Brugada patients. Hence, further studies involving both repolarization and depolarization analyses should be conducted to improve the discriminative capability of potential markers derived from the ECG.

## Conclusions

Symptomatic and asymptomatic patients with Brugada syndrome studied in this work have shown significantly different ventricular depolarization dynamics during exercise and mostly during recovery. Such differences are mainly observed through the proposed novel depolarization indices, associated with the second half and/or terminal part of the QRS complex. These findings may be useful to improve risk stratification for malignant arrhythmic events, specially in individual asymptomatic patients. Since the obtained results came from a retrospective data, further work is warranted to evaluate the proposed indices in a prospective study, with a larger patient population, and to assess the added value of a combined analysis of cardiac depolarization and repolarization parameters, together with different clinical factors and genetic status.

## Supporting information

S1 Table Mean values of the ECG markers evaluated in this study. (PDF)

S2 Table Raw values of the different markers extracted from ECG signals. (XLSX)
