# Supplementary material for: Dynamic changes in ventricular depolarization during exercise in patients with Brugada syndrome
Source: PLoS One. 2020 Mar 3;15(3):e0229078. doi: 10.1371/journal.pone.0229078 (PMC7053736; doi:10.1371/journal.pone.0229078)
Supplement: S1 Table — (PDF) [file pone.0229078.s001.pdf]

S1 Table. Mean ± SEM values of the depolarization markers evaluated in the study.

Marker	Asymptomatic			Symptomatic		
	EX_{BEG}	EX_{MAX}	RE_{END}	EX_{BEG}	EX_{MAX}	RE_{END}
A_R (uV)						
V1	147.8 ± 11.6	230.6 ± 41.7	167.7 ± 13.1	126.7 ± 21.1	156.7 ± 21.7	146.5 ± 24.2
V2	331.7 ± 30.2	274.5 ± 24.4	321.2 ± 27.5	399.5 ± 72.9	342.9 ± 49.9	371.8 ± 63.4
V3	667.7 ± 42.7	563.2 ± 42.7	654.9 ± 27.5	741.5 ± 72.9	714.9 ± 49.9	749.0 ± 63.4
A_S (uV)						
V1	-757.2 ± 44.2	-698.8 ± 42.9	- 829.2 ± 57.5	-643.2 ± 73.4	-654.9 ± 77.9	-686.4 ± 84.9
V2	-922.2 ± 75.4	-931.6 ± 77.1	-1084.7 ± 91.6	-704.9 ± 103.6	-778.5 ± 116.3	-797.2 ± 110.7
V3	-899.2 ± 57.1	-1138.8 ± 62.6	-1130.3 ± 68.6	-711.5 ± 88.8	-970.8 ± 98.9	-920.6 ± 100.7
U_R (Uv/ms)						
V1	10.6 ± 0.7	16.5 ± 2.5	12.0 ± 0.9	9.5 ± 1.4	13.0 ± 1.5	11.1 ± 1.6
V2	21.1 ± 1.7	20.8 ± 1.4	21.4 ± 1.6	24.6 ± 3.9	23.3 ± 2.9	23.1 ± 3.5
V3	37.5 ± 2.3	39.9 ± 2.5	40.0 ± 2.4	41.0 ± 4.2	45.9 ± 4.8	43.5 ± 4.7
D_R (uV/ms)						
V1	-50.1 ± 2.6	-50.9 ± 3.2	-56.2 ± 3.4	-45.9 ± 4.6	-46.1 ± 4.8	-49.9 ± 5.3
V2	-71.6 ± 5.3	-70.1 ± 5.2	-80.4 ± 6.0	-66.4 ± 7.9	-63.9 ± 6.8	-69.8 ± 7.1
V3	-92.2 ± 4.4	-102.0 ± 4.9	-104.9 ± 4.8	-87.6 ± 5.8	-103.7 ± 7.6	-100.2 ± 6.9
U_S (uV/ms)						
V1	42.1 ± 2.3	46.5 ± 2.9	48.4 ± 2.9	40.6 ± 4.0	42.9 ± 4.4	45.4 ± 4.7
V2	51.7 ± 2.9	51.7 ± 3.2	60.7 ± 3.8	43.9 ± 4.1	44.9 ± 4.5	48.3 ± 4.5
V3	45.2 ± 2.2	52.1 ± 2.4	54.9 ± 2.8	36.9 ± 3.9	44.7 ± 4.2	44.1 ± 4.3
QRSd (ms)	110.1 ± 1.9	116.5 ± 2.5	110.3 ± 1.6	109.1 ± 2.4	110.7 ± 4.1	111.1 ± 3.0
